# Eradication of common pathogens at days 2, 3 and 4 of moxifloxacin therapy in patients with acute bacterial sinusitis

**DOI:** 10.1186/1472-6815-6-8

**Published:** 2006-04-28

**Authors:** Horacio Ariza, Ramon Rojas, Peter Johnson, Richard Gower, Alice Benson, Janet Herrington, Renee Perroncel, Peter Pertel

**Affiliations:** 1 H.Z.G.A. "Mi Pueblo", Buenos Aires, Argentina; 2 Centro Privado de Cardiología, Tucumán, Argentina; 3 ENT Consultants of Winchester, Inc., Winchester, VA, USA; 4 Rockwood Clinic, Spokane, WA, USA; 5 Bayer HealthCare Pharmaceuticals, West Haven, CT, USA

## Abstract

**Background:**

Acute bacterial sinusitis (ABS) is a common infection in clinical practice. Data on time to bacteriologic eradication after antimicrobial therapy are lacking for most agents, but are necessary in order to optimize therapy. This was a prospective, single-arm, open-label, multicenter study to determine the time to bacteriologic eradication in ABS patients (maxillary sinusitis) treated with moxifloxacin.

**Methods:**

Adult patients with radiologically and clinically confirmed ABS received once-daily moxifloxacin 400 mg for 10 days. Middle meatus secretion sampling was performed using nasal endoscopy pre-therapy, and repeated on 3 consecutive days during treatment. Target enrollment was 30 bacteriologically evaluable patients (pre-therapy culture positive for *Streptococcus pneumoniae*, *Haemophilus influenzae *or *Moraxella catarrhalis *and evaluable cultures for at least Day 2 and Day 3 during therapy visits), including at least 10 each with *S. pneumoniae *or *H. influenzae*.

**Results:**

Of 192 patients enrolled, 42 were bacteriologically evaluable, with 48 pathogens isolated. Moxifloxacin was started on Day 1. Baseline bacteria were eradicated in 35/42 (83.3%) patients by day 2, 42/42 (100%) patients by day 3, and 41/42 (97.6%) patients by day 4. In terms of individual pathogens, 12/18 *S. pneumoniae*, 22/23 *H. influenzae *and 7/7 *M. catarrhalis *were eradicated by day 2 (total 41/48; 85.4%), and 18/18 *S. pneumoniae *and 23/23 *H. influenzae *were eradicated by day 3. On Day 4, *S. pneumoniae *was isolated from a patient who had negative cultures on Days 2 and 3. Thus, the Day 4 eradication rate was 47/48 (97.9%). Clinical success was achieved in 36/38 (94.7%) patients at the test of cure visit.

**Conclusion:**

In patients with ABS (maxillary sinusitis), moxifloxacin 400 mg once daily for 10 days resulted in eradication of baseline bacteria in 83.3% of patients by Day 2, 100% by Day 3 and 97.6% by Day 4.

## Background

The increasing prevalence of bacterial resistance in acute bacterial sinusitis (ABS) is of concern [1]. In particular, penicillin resistance, macrolide resistance and trimethoprim-sulfamethoxazole resistance in *Streptococcus pneumoniae*, and β-lactamase production in *Haemophilus influenzae *and *Moraxella catarrhalis*, can present clinical problems in regions where their prevalence is high [1]. Worldwide, penicillin resistance has been reported in about 20–30% of clinical isolates of *S. pneumoniae *[2-5], while approximately 15–40% of isolates of *H. influenzae *produce β-lactamase conferring resistance to ampicillin [3-5]. High rates of β-lactamase production (over 90%) and associated resistance have also been reported in *M. catarrhalis *[3,5].

*In vitro *surveillance data demonstrate excellent activity of the fluoroquinolone moxifloxacin against ABS pathogens, including strains resistant to other antimicrobial classes [5-7]. In addition, moxifloxacin shows good penetration into sinus tissue, achieving concentrations in maxillary sinus mucosa that range from 5- to 30-fold higher than the minimum inhibitory concentration required to inhibit growth of 90% (MIC_90_) of *S. pneumoniae *isolates 2 to 36 hours after the last 400 mg dose in a 5-day course [8].

Currently, in the United States moxifloxacin 400 mg once daily for 10 days is approved for the treatment of ABS. This reflects the traditional practice of giving antimicrobial therapy for ABS for 10–14 days. However, new antimicrobials are increasingly being investigated at shorter treatment durations. For example, in previous studies in ABS, moxifloxacin 400 mg once daily for 7 days had excellent clinical efficacy, with clinical and bacteriologic success rates ranging from approximately 92% to 97% [9-11]. Also, moxifloxacin 400 mg once daily for 7 days was clinically and bacteriologically superior to cefuroxime axetil 250 mg twice daily for 10 days [11]. In a large post-marketing study of moxifloxacin in ABS (N = 2405), 96% of patients had improved by Day 5 and the clinical success rate by Day 10 was 97% [12]. Studies with other antibiotics have confirmed that shorter courses of therapy (3–5 days) produce comparable clinical and bacteriological outcomes to longer courses [13-16].

Bacteriologic eradication data provide the most accurate indication of antimicrobial effect in ABS. However, these data are rarely collected and then only at the start and end of therapy. In order to optimize the dosing duration, data on the dynamics of bacteriologic eradication are required, but these data are generally lacking for most antimicrobial agents.

The objective of this study was to determine the time to bacteriologic eradication following moxifloxacin therapy in patients with ABS and positive pre-therapy bacterial cultures.

## Methods

### Study design

This was a prospective, single-arm, open-label, multicenter study conducted in adult patients with radiologically and clinically confirmed ABS. Patients received once-daily moxifloxacin 400 mg for 10 days; however, additional or alternative antimicrobial therapy was permitted if the patient was a failure following treatment with moxifloxacin. All patients underwent middle meatus secretion sampling by means of nasal endoscopy pre-therapy, and the procedure was repeated on 3 consecutive days during treatment (Days 2, 3 and 4). Topical nasal medications were not permitted within 4 hours before endoscopy. Preparation of the nasal fossa, endoscopic sampling and transport of the sample to the local microbiology laboratory were performed according to local practices at each center. Antimicrobial susceptibility testing was performed at each center using standard broth microdilution methods [17]. The study was conducted in accordance with the Declaration of Helsinki and the protocol approved by the ethical review board of each participating center.

### Patient population

Subjects were males or females aged ≥18 years who had a clinical diagnosis of ABS with signs and symptoms present for ≥7 days but <28 days. ABS signs or symptoms were defined as follows: evidence of air-fluid levels and/or opacification on radiographic paranasal sinus film (Waters' view) or limited CT scan; purulent secretions obtained pre-therapy via middle meatus secretion sampling using nasal endoscopy; and the presence of at least one major and one minor symptom. Major symptoms were purulent anterior or posterior nasal discharge, and unilateral moderate or severe facial pain or malar tenderness; minor symptoms were cough or frequent throat clearing, frontal headache, halitosis, and fever (oral ≥38.0°C/100.4°F, tympanic ≥38.5°C/101.2°F). All women of childbearing potential enrolled in the study had to have a negative urine pregnancy test and practice adequate contraceptive use. Written informed consent was obtained prior to enrollment.

Patients were excluded from the study if they had a history of chronic sinusitis or symptoms of allergic rhinitis, previous or concomitant antimicrobial treatment, serious/systemic infection, immunological impairment or terminal illness, use of topical nasal or systemic steroids (unless the dose had been stable for >4 weeks), or known hypersensitivity to study medication. Other exclusion criteria were pregnancy or breast feeding, risk of possible drug interactions, severe liver disease, renal impairment, uncorrected hyperkalemia, QT_c _prolongation, quinolone-associated tendinopathy, previous inclusion in this study or investigational drug use in the last 30 days.

### Efficacy evaluation

The primary efficacy variable was the proportion of patients with bacteriologic eradication of pre-therapy pathogens from middle meatus secretions on Days 2, 3, and 4 in bacteriologically evaluable patients, i.e. the per-protocol population. Patients in the per-protocol population had to meet the study inclusion criteria, receive study drug on Days 1–4 (except in the case of clinical failure where two doses were required); have a positive baseline culture for at least one of the following: *S. pneumoniae*, *H. influenzae*, or *M. catarrhalis*; have evaluable culture specimens obtained on Day 2 and Day 3 of treatment; receive no other antimicrobial therapy from 7 days prior to enrollment through day 4 of study drug administration unless assessed as a clinical or bacteriological failure; and have no protocol violation that would affect treatment efficacy or evaluation.

Clinical efficacy was evaluated at the test of cure visit (0–3 days post-treatment [Days 10–13]). Clinical success was defined as resolution or improvement in the signs and symptoms of ABS such that no further antimicrobial therapy was required; clinical failure was defined as lack of improvement or worsening of ABS symptoms such that further antimicrobial therapy was required. The subset of patients deemed clinically evaluable, i.e. the clinical efficacy population, had to meet the inclusion criteria for the study and receive at least 80% of the prescribed study drug doses (except in the case of clinical failure where two doses were required), have a valid clinical evaluation available at the test of cure visit and no protocol violation that would affect treatment efficacy or evaluation. Again, no other antimicrobial therapy could be administered from 7 days prior to enrollment until through the test of cure visit unless the patient was assessed as a clinical or bacteriological failure.

### Safety evaluation

Clinical adverse events and laboratory data among all moxifloxacin-treated patients were recorded. Adverse events were classified according to the MedDRA glossary.

### Statistical analysis

The planned sample size of 200 patients was based on obtaining 30 valid per-protocol patients, including at least 10 each with *S. pneumoniae *or *H. influenzae*. Assuming an 80% eradication rate on Day 3 of therapy, the associated 95% confidence interval (CI) would be 65%-95%; these limits were considered to provide sufficient precision. Two-sided confidence intervals were calculated by exact methods using SAS^® ^software.

## Results

### Patient population

Eight sites in Argentina enrolled 65 patients and 22 sites in the United States enrolled 127 patients, giving a total of 192 enrolled patients. The majority of patients (184/192, 95.8%) completed the study as planned. Eight patients discontinued prematurely: 3 patients did not comply with the protocol, 2 withdrew due to adverse events and 2 due to insufficient therapeutic effect, and 1 was lost to follow-up.

Of the 192 patients enrolled, 42 were bacteriologically evaluable (per-protocol population), with 48 pathogens isolated. Of these, 7 sites in Argentina enrolled 24 patients and 13 sites in the United States enrolled 18 patients. Only 1 patient who had a positive bacterial culture at baseline was excluded from the per-protocol population; this was due to the duration of the current episode of sinusitis being too short (6 days). All 42 patients in the per-protocol evaluation were bacteriologically evaluable at Days 2, 3 and 4.

All patients in the per protocol population had maxillary sinusitis. As assessed by the investigator, one patient had a mild infection, 32 patients had moderate infections, and nine had severe infections. The baseline demographic and clinical characteristics of these patients are shown in Table 1.

**Table 1:** Baseline demographic and clinical characteristics (per-protocol population)

Characteristic	Per-protocol population (N = 42)
Sex, n male (%)	13 (31.0%)
Race, n (%)	
white	34 (81.0%)
hispanic	6 (14.3%)
black	2 (4.8%)
Mean age at enrollment, years ± SD (range)	41.0 ± 15.2 (18.0–76.0)
Mean weight, kg ± SD (range)	74.8 ± 17.4 (41.0–113.4)
Mean BMI ± SD (range)	27.0 ± 6.2 (18.0–46.9)
Location of infection, n (%)	
left	13 (31.0)
right	13 (31.0)
bilateral	16 (38.1)
Severity of infection, n (%)	
mild	1 (2.4)
moderate	32 (76.2)
severe	9 (21.4)
Median duration of infection, days (range)	11 (7–21)

SD, standard deviation

Table 2 presents the baseline ABS signs and symptoms in the per-protocol population. With the exception of halitosis, most patients had moderate to severe signs and symptoms. The most common presenting symptoms were purulent nasal discharge and facial pain. Fever was present in only 1 patient.

**Table 2:** Baseline severity of ABS signs and symptoms (per-protocol population, N = 42)

Sign/symptom	Patients (%) with severity of:			
	None	Mild	Moderate	Severe
Purulent nasal drainage	1 (2.4)	2 (4.8)	17 (40.5)	22 (52.4)
Facial pain	3 (7.1)	4 (9.5)	19 (45.2)	16 (38.1)
Frontal headache	5 (11.9)	5 (11.9)	15 (35.7)	17 (40.5)
Throat clearing	6 (14.3)	10 (23.8)	20 (47.6)	6 (14.3)
Cough	9 (21.4)	9 (21.4)	12 (28.6)	12 (28.6)
Malar tenderness/pain	10 (23.8)	9 (21.4)	15 (35.7)	8 (19.0)
Halitosis	14 (33.3)	10 (23.8)	14 (33.3)	4 (9.5)

Of the 48 causative organisms identified from pre-treatment cultures on Day 1 in the per-protocol population, there were 23 *H. influenzae*, 18 *S. pneumoniae *and 7 *M. catarrhalis *isolates. Six patients had 2 organisms isolated; in 5 patients these were *H. influenzae *and *S. pneumoniae*; 1 patient had *H. influenzae *and *M. catarrhalis*. No strains were typed. Susceptibility to moxifloxacin was determined for 45 isolates (3 *H. influenzae *isolates were not tested). The moxifloxacin MIC_90 _(with MIC range) was 0.06 mg/L (0.015–0.06 mg/L) for *H. influenzae*, 0.250 mg/L (0.06–0.25 mg/L) for *S. pneumoniae *and 0.06 mg/L (0.03–0.06 mg/L) for *M. catarrhalis*. For azithromycin susceptibility the corresponding figures were 4.0 mg/L (0.5–8.0 mg/L) for *H. influenzae *and 8.0 mg/L (0.125–16.0 mg/L) for *S. pneumoniae*. For clarithromycin susceptibility the figures were 16.0 mg/L (2.0–32.0 mg/L) for *H. influenzae *and 8.0 mg/L (0.125–8.0 mg/L) for *S. pneumoniae*. For penicillin, the MIC_90 _against *S. pneumoniae *was 4.0 mg/L (0.008–8 mg/L). All of the 7 *M. catarrhalis *strains and 7 of the 20 *H. influenzae *strains tested (35%) were β-lactamase-positive.

### Primary efficacy outcome

Baseline bacteria were eradicated in 35/42 patients (83.3%; 95% CI: 68.6, 93.0) by Day 2, 42/42 patients (100%; 95% CI: 91.6, 100) by day 3, and 41/42 patients (97.6%; 95% CI: 87.4, 99.9) by Day 4.

**Figure 1:**
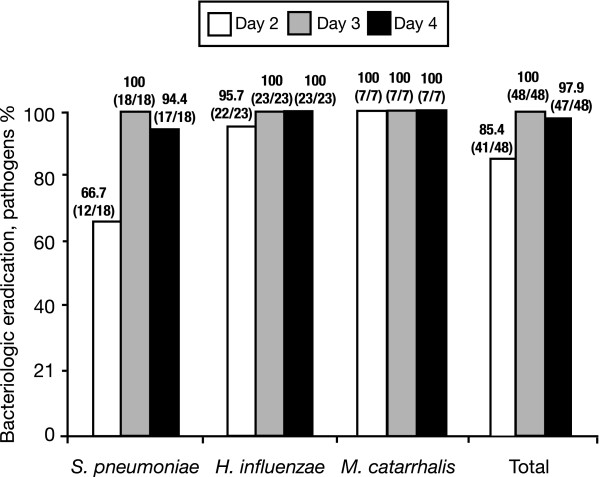
**Bacterial eradication by pathogen**. Proportion of baseline ABS pathogens eradicated from middle meatus secretions at Days 2, 3, and 4 of moxifloxacin therapy.

In terms of individual pathogens, 12/18 *S. pneumoniae*, 22/23 *H. influenzae *and 7/7 *M. catarrhalis *isolates were eradicated by Day 2 (total 41/48; 85.4%) (Figure 1). Susceptibility data were available for 3 of the 6 *S. pneumoniae *isolates and the 1 *H. influenzae *isolate that persisted; all 4 isolates remained susceptible to moxifloxacin, with MICs identical to those recorded at baseline.

By Day 3, 18/18 *S. pneumoniae *isolates and 23/23 *H. influenzae *isolates were eradicated (Figure 1). On Day 4, *S. pneumoniae *was isolated from a patient who had negative cultures on Days 2 and 3 (Figure 1). This patient was a clinical success at the test of cure visit. The MIC for the remaining *S. pneumoniae *isolate was 0.125 mg/L, which was the same as that recorded at baseline.

### Clinical outcome

Assessment of clinical outcome was outside the specified time of the test of cure assessment for 4 patients in the per-protocol group, and these were excluded from the clinical efficacy analysis. In the per-protocol group 36/38 (94.7%; 95% CI: 82.3, 99.4) patients were clinical successes at the test of cure visit. The remaining 2 patients were clinical failures at the test of cure visit, but had shown clinical improvement at Days 3 and 4 (during therapy). The baseline pathogens for these 2 patients, (1 *S. pneumoniae *and 1 *M. catarrhalis*) were eradicated at Day 4.

A total of 162 patients were included in the clinical efficacy population. The most common reason for exclusion from this population was violation of the time schedule (11 patients, i.e. test of cure visit outside Days 0–3 post-treatment). At the test of cure visit 150/162 (92.6%; 95% CI: 87.4, 96.1) patients were clinical successes. Of the 150 patients classes as clinical successes, 72 (48%) were totally free of symptoms and 78 (52%) had improvements in symptoms. The 12 patients classified as treatment failures constituted a heterogeneous group, consisting of 7 males and 5 females and ranging in age from 24–79 years (mean: 46.2 years). Eight (67%) were white, 2 were black, 1 was Asian, and 1 Hispanic. The severity of infection at baseline was mild in 1 patient, moderate in 6 patients, and severe in 5 patients; 3 had bilateral sinusitis. All 12 patients were treated in the United States.

The safety (intent-to-treat) population included all patients who received at least one dose of study drug (n = 187). In the safety population 168/187 (89.8%; 95% CI: 84.6, 93.8) were clinical successes at test of cure.

### Safety

Because of missing information at one of the study sites that would not permit confirmation of data, 5 of the 192 enrolled patients were not included in the safety population. All 5 patients appear to have completed 10 days of study drug, and only one patient had adverse events (mild nausea and mild pyrosis). Of the 187 patients valid for the safety analysis, 64 (34%) experienced at least one adverse event and 29 (16%) reported at least 1 possibly or probably drug-related adverse event. The most common adverse events considered to be possibly or probably drug-related are listed in Table 3. Treatment was generally well tolerated. There were 2 withdrawals due to adverse events: one due to an allergic reaction to study drug and one due to an incarcerated inguinal hernia. There were no drug-related serious adverse events although one patient developed an inguinal hernia and an ileus that were both assessed as serious and not related to study drug.

**Table 3:** Drug-related adverse events occurring in at least 2% of patients (safety population)

Adverse event, n (%)	Safety population (N = 187)
Nausea	10 (5.3)
Dry mouth	4 (2.1)
Diarrhea	5 (2.7)
Irritability	4 (2.1)

## Discussion

This study showed high rates of bacteriologic eradication of common ABS pathogens with moxifloxacin, 400 mg once daily, in the first 2–4 days of therapy. In addition, clinical success was achieved in a high proportion of patients.

These results suggest that the duration of therapy could be shortened without compromising clinical efficacy. However, the relationship between bacteriologic eradication and clinical success in this study was not complete. One patient with *S. pneumoniae *cultured at Day 4 was a clinical success at Day 10–13, while 2 patients with bacteriologic eradication at Day 4 were clinical failures at the test of cure visit. Furthermore, this study was not designed to test whether a shortened moxifloxacin course is effective in the treatment of ABS.

In the case of the 1 patient who had a recurrence of *S. pneumoniae *at Day 4, it is unknown whether this was a true recurrence or a new infection. Also, failure to culture an organism obtained from any microbiologic sample is a good, but imperfect, indication of bacteriologic eradication. Direct endoscopic nasal cultures were obtained in this study, rather than sinus puncture cultures. When compared to sinus puncture cultures, endoscopic cultures from the middle meatus have a sensitivity of 71.4% and a specificity of 53.1% to detect any pathogen associated with sinusitis [18]. However, for the detection of *S. pneumoniae*, *H. influenzae *and *M. catarrhalis *the sensitivity and specificity improve to 85.7% and 90.6%, respectively, and the positive predictive and negative predictive values are 80.0% and 93.5%, respectively [18,19]. This is consistent with the results with a recent meta-analysis [19], which found that for known pathogens the sensitivity and specificity of endoscopically directed culture in acute bacterial sinusitis were 80.9% and 90.5%, respectively, and the positive predictive and negative predictive values were 82.6% and 89.4%, respectively. It could be that the negative cultures on Days 2 and 3 for the one patient with *S. pneumoniae *culture at Day 4 represents either an inability to detect low colony counts or sampling error.

Clinical success rates after 10 days of moxifloxacin therapy were high for all patient populations analyzed; 94.7%, 92.6% and 89.8% for the per-protocol, clinical efficacy and intent-to-treat populations, respectively. These results are consistent with those predicted by the Sinus and Allergy Health Partnership (SAHP) guidelines using the Poole Therapeutic Outcome Model for fluoroquinolones (90–92% potential for clinical success) [1]. However, moxifloxacin has greater *in vitro *activity against *S. pneumoniae *than other fluoroquinolones indicated for use in ABS, such as levofloxacin and gatifloxacin [20,21]. Thus, the results of this study cannot be extrapolated to other members of the class for this pathogen. To our knowledge, only one other report has been made on time to sinus sterilization with antibiotic therapy [22]. Of the 12 patients enrolled, only 10 were clinically evaluable and the organisms isolated included *S. pneumoniae *(4 isolates), *S. aureus *(2 isolates), coagulase-negative staphylococci (2 isolates), viridans group streptococci (1 isolate), and *Enterobacter aerogenes *(1 isolate). For the *S*.*pneumoniae*, the median time to sinus sterilization was 50 hours (range 24 to 74 hours); however, no firm conclusions can be drawn from such a small sample number.

The SAHP guidelines emphasize that pharmacodynamic and pharmacokinetic properties are an important consideration in the choice of antibiotic therapy for ABS [1]. Comparative data regarding the time to bacterial eradication would be useful to enable the development of rational, optimized dosing schedules in ABS.

## Conclusion

This study provided valuable data on the speed of eradication with moxifloxacin in patients with ABS and positive pre-therapy bacterial cultures. Moxifloxacin therapy (400 mg once daily for 10 days) resulted in eradication of baseline bacteria in 83.3% of patients by Day 2, 100% by Day 3 and 97.6% by Day 4. Early eradication of ABS pathogens with moxifloxacin indicates the potential for optimization of therapy duration with maintained high efficacy.

## Competing interests

HA, RR, PJ and RG declare that they have no competing interests. AB, JH, RP and PP are employees of Bayer HealthCare Pharmaceuticals.

## Authors' contributions

HA, RR, PJ and RG participated in patient recruitment, clinical assessment, data interpretation and contributed to manuscript development. AB performed the statistical analysis and reporting and assisted in the drafting of this manuscript. RP participated in the study design, coordination and reporting and helped to draft the manuscript. JH was responsible for microbiological analyses. PP participated in the coordination and reporting of the study, data interpretation, and drafting the manuscript. All authors read and approved the final manuscript.

## Participating investigators and institutions

**Argentina: **A. Galgano, Policlínico Bancario Pulmonar Lab, Buenos Aires; H. Ariza, Hosp. Municipal de Agudos "Mi Pueblo" Buenos Aires; V. Criquet, Sanatorio Privado María Mater, Buenos Aires; S. Lupo, Instituto Caici Santa Fé; G. Ambasch, Nuevo Hospital San Roque, Córdoba; HH Altieri, Hospital Centro de Salud Zenon Santillan, Tucumán; R. Rojas, Centro Privado de Cardiología, Tucumán; **USA: **D. Britt, Longmont Medical Research Network, Longmont, CO; J. Hohengarten, Colorado Otolaryngology Associates, Colorado Springs, CO; E. Lane, Connecticut Sinus Center, Bridgeport, CT; S. Singh, SARC Research Center, Fresno, CA; J. Sullivan, Parkway Medical Center, Birmingham, AL; N. Kao, Allergic Disease & Asthma Center, Greenville, SC; J. Baker, Allergy, Asthma & Dermatology Research Center, LLC, Lake Oswego, OR; J. Adelglass, Research Across America, Carrollton, TX; D. Blaine, Charlottesville Medical Research, Charlottesville, VA; J. Bundy, Mercy Health-Canadian County, Yukon, OK; M. Collins, Clinical Research Consultants, Inc., Hoover, AL; M. DiGiovanna, DiGiovanna Family Care Center, North Massapequa, NY; P. Johnson, ENT Consultants of Winchester, Inc., Winchester, VA; B. Lansford, NEA Clinic, Jonesboro, AR; D. McNeil, Optimed Research, Columbus, OH; R. Nielsen, Ear, Nose, & Throat Center of Salt Lake, Salt Lake City, UT; J. Parker, Highland Clinic, Shreveport, LA; W. Preston, Jackson Clinic, Jackson, TN; B. Rankin, University Clinical Research – DeLand, DeLand, FL; R. Sterling, Sterling Ear, Nose, & Throat, PA, Orangeburg, SC; M. Tarpay, Allergy, Asthma & Clinical Research Center, Oklahoma City, OK; R. Gower, Rockwood Clinic, Spokane, WA.

## Pre-publication history

The pre-publication history for this paper can be accessed here:


